# Research on the spatial layout optimization strategy of Huaihe Road Commercial Block in Hefei city based on space syntax theory

**DOI:** 10.3389/fncom.2022.1084279

**Published:** 2023-01-10

**Authors:** Qinghua Zhou, Yiran Zheng

**Affiliations:** School of Architecture and Urban Planning, Anhui Jianzhu University, Hefei, Anhui, China

**Keywords:** space syntax theory, commercial block, spatial layout, optimization strategy, urban renewal

## Abstract

Commercial block not only serves as a public space for the consumption, entertainment, and recreation of residents but also witnesses the history of urban commercial development. With the urban development and the improvement of people’s living standards, most commercial blocks are faced with such problems as traffic congestion, simple commercial form, and unreasonable spatial layout. By taking the commercial block of Huaihe Commercial Pedestrian Street as an example and combining the axis and viewshed analysis method of space syntax theory, this article has analyzed the space and quantified the relevant data to analyze the spatial layout relationships of commercial blocks. As the outcomes, this article summarizes the strategies for optimizing the traffic space, scenic space, and commercial space of commercial blocks, hoping to facilitate the commercial block space layout optimization in the era of stock.

## 1. Overview of the research

### 1.1. Research background

As the capital city of Anhui Province, Hefei is situated in the central region of East China. Linking with Shanghai, Nanjing, Hangzhou, Wuhan, and other important cities, it gradually becomes a bridge and bond between East China and Central China. After being included in the Yangtze River Delta urban cluster, Hefei has a clear urban orientation. Its population scale has been increasing. It sees rapid economic development. These factors have boosted urban development and renovation at the same time.

Urban planning approaches have evolved from increment planning to stock planning. In this process, more and more problems in old urban areas, such as high building density, dense population, and great traffic pressure, are unveiled. Commercial blocks also face the same problems. Huaihe Road Commercial Block is located in the east of the old town of Hebei. It is the city’s most representative historical commercial pedestrian street. Starting from Huancheng Road in the east and extending westwards to Suzhou Road, this pedestrian street is 920 m long and 22 m wide. Huaihe Road Commercial Block is a key old town renovation project in the process of urban renewal. It is urgent to solve such problems as traffic congestion in rush hours, simple and low-quality commercial forms, and low-quality spatial quality of commercial blocks.

### 1.2. Research object

**FIGURE 1:**
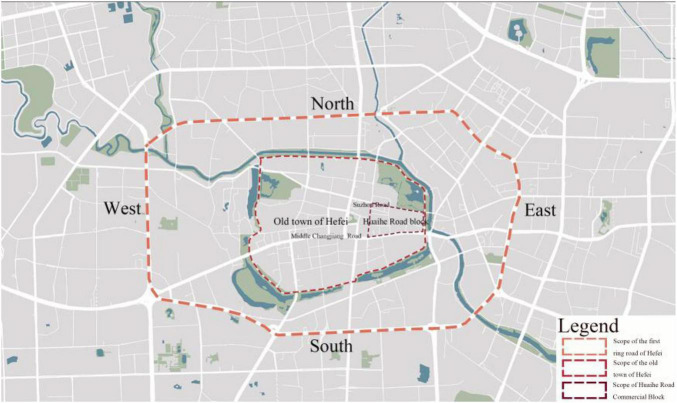
Scope of the old town of Hefei.

This article takes the spatial layout of Huaihe Road Commercial Block as the research object. Analysis and research have been performed on the block in three aspects, including traffic space, landscape space, and commercial space. The research of this article started with the discussion and analysis of urban location planning and block spatial layout. The research scope is shown in Figure 1. At first, the old town was analyzed from the perspective of urban location, thereby obtaining the traffic accessibilities and road connections of the block in the old town of Hefei. Then, the block was analyzed, thereby learning about its traffic accessibilities, road connections, and spatial identifications.

## 2. Research methods

### 2.1. Space syntax theory

The space syntax theory was pioneered in the late 1970s by Professor Bill Hillier, a scholar at the University of London (Hillier, 1996). The algorithm of this theory is the quantitative rational analysis of spatial relations based on topological relations. This theory was formulated based on topological relations. By performing quantitative analysis of spatial layouts, it is used to investigate the relations between the spatial organization and human society (Hillier and Sheng, 2004). In recent years, urban renovation actions have boosted block renovation projects. This fact has been supported by related theoretical articles. For example, the spatial relation between the commercial pedestrian street and commercial district, the location of commercial pedestrian streets, and the layout of road intersections of commercial pedestrian streets with urban trunk roads in the Planning Application of Space Syntax in Urban Commercial Pedestrian Street written by Tang Jing are the rational research results based on the space syntax analysis.

### 2.2. Application of ArcGIS software

The spatial layout of commercial forms in a block is a significant factor in analyzing the commercial space layout of the block (Wang et al., 2008). ArcGIS software can provide corresponding help. Road network data and point of interest (POI) data of a block are obtained (Chi et al., 2016).

### 2.3. Indicators of the space syntax theory

The space syntax theory refers to an analysis method based on spatial topological relations, whose indicators are cited as the major criteria to reflect spatial relations. The spatial layout relations of Huaihe Road Commercial Block were selected as the research object of this article. The following five indicators are chosen for the quantitative analysis of spatial relations (refer to Table 1).

**TABLE 1:** Indicators of the space syntax theory.

Indicator	Property	Indication
Integration	Tie	Indicate the degree of tie and accessibility of system nodes with other nodes. Higher integration corresponds to greater degree of tie and accessibility of system nodes.
Depth	Accessibility	Indicate the topological distance between two adjacent nodes. The number of spatial turns is the depth value between the two nodes (Liu and Jiang, 2012). Smaller depth value between two nodes corresponds to better accessibility.
Connection	Connectivity	Indicate the number of spatial connections in the system node space. Larger number of connections between system space and other node spaces corresponds to larger connection value and better system space tie.
Selection	Selectivity	Indicate the number of times the system node space appears on the shortest topological path. For the selection of the shortest path, higher selection corresponds to better traffic attributes and potentials (Xing and Guo, 2022).
Intelligibility	Identifiability	Indicate the relationship between local space and overall space and indicate whether the overall space can be judged through local space. Higher intelligibility corresponds to the people’s stronger perception toward space.

## 3. Analysis of spatial layout of Huaihe Road Commercial Block based on space syntax

The spatial relationship analysis method of syntax theory is used to analyze the basic characteristics of research objects (Hillier and Hanson, 1984). In this article, the relevant data of Huaihe Road Commercial Block, such as road network data and point of interest (POI) data points, were obtained through a map platform and field studies (Jeong and Ban, 2011). In summary, axis analysis was conducted for the road network relations of the old town of Hefei through modeling. In addition, an axis model and viewshed model were created, respectively, for the road network and building profiles of the commercial block for targeted analysis.

### 3.1. Axis analysis of the old town of Hefei

#### 3.1.1. Depth of the old town of Hefei

**FIGURE 2:**
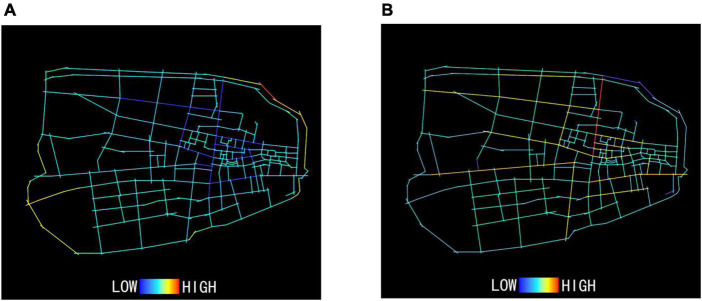
Depth analysis of the old town of Hefei **(A)**. Connection analysis of the old town of Hefei **(B)**.

As shown in Figure 2A and Table 2, the overall color of the axis of the old town is cool with a mean depth value of 1,376.25, while the axis of some urban roads in the periphery of the old town is warm with larger depth value and lower accessibility. The overall color of the axis of Huaihe Road Commercial Block is cool and the axis depth value is 1,000, lower than the mean depth value of the old town, indicating a smaller axis depth but stronger accessibility. In the process of field investigation, it was found that the construction of subway and bus stations around the block would effectively improve the accessibility of the block’s east and west exits and entrances and make the exits and entrances more important in the overall block space.

**TABLE 2:** Axis depth and connection of the old town of Hefei.

Indicator name	Depth	Connection
Mean value	1,376.25	3.52
Minimum value	905	1
Maximum value	2,482	11
Standard deviation	238.96	1.80

#### 3.1.2. Connection to the old town of Hefei

As shown in Figure 2B and Table 2, the mean spatial connection of the old town is 3.52 and most of the long axes in the old town are in warm color because the urban road space connects the rest of the branch roads. Roads around Huaihe Road Commercial Block, such as Suzhou Road and Middle Changjiang Road, are of long axes in warm color with a connectivity of 8–11, higher than the mean connection of the old town. These roads are the trunk ones connecting the old town with the block. Better road connections indicate a stronger tie between the block and other areas of the city.

### 3.2. Axis analysis of Huaihe Road Commercial Block

#### 3.2.1. Axis depth of Huaihe Road Commercial Block

**FIGURE 3:**
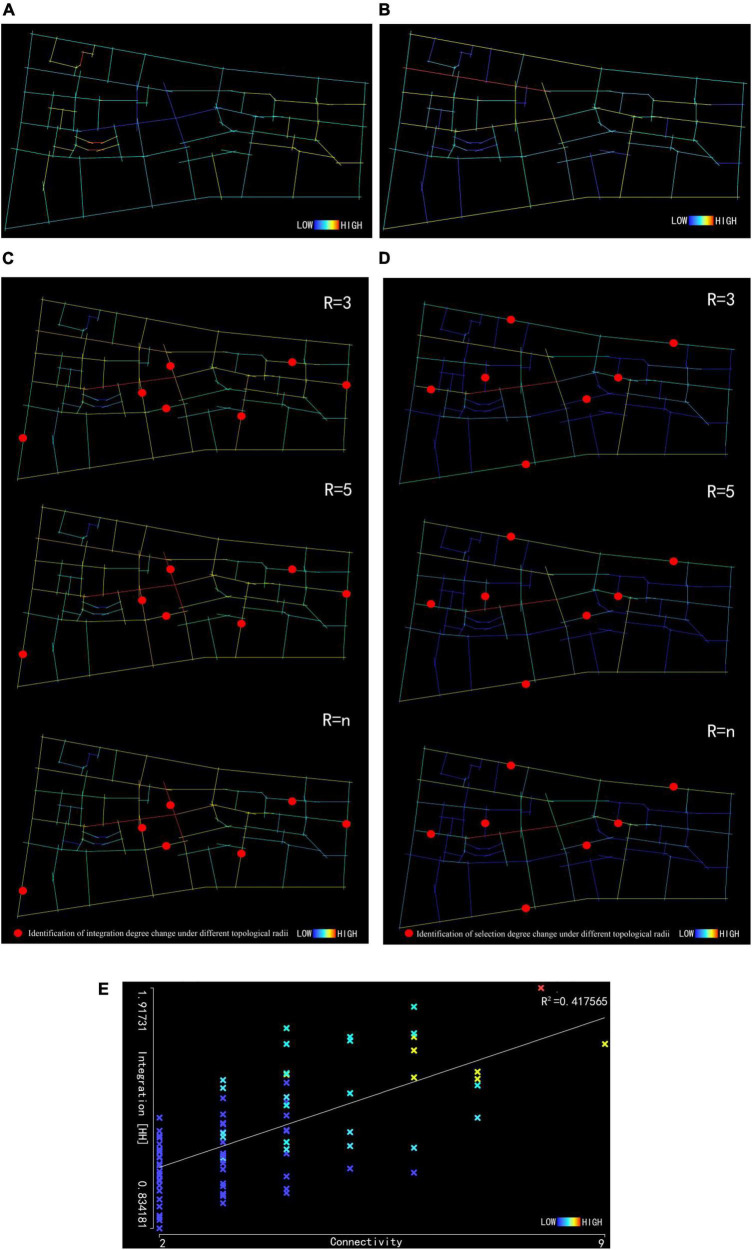
Axis depth analysis of Huaihe Road Commercial Block **(A)**. Axis connection analysis of Huaihe Road Commercial Block **(B)**. Axis integration analysis of Huaihe Road Commercial Block **(C)**. Axis selection analysis of Huaihe Road Commercial Block **(D)**. Axis intelligibility analysis of Huaihe Road Commercial Block **(E)**.

As shown in Figure 3A and Table 3, the depth of the block is 391.25, slightly lower than the mean depth value of the old town. The block axis color is typically cool, indicating that the block has a smaller road network depth but stronger spatial accessibility. As the main street, the commercial pedestrian street shows a cool overall color with a depth range of 282–339, indicating stronger accessibility. After field investigations, it was also found that the main street of Huaihe Road plays an important role in the block’s road network. The surrounding residents and tourists are more willing to hang out on the main street, significantly affecting the pedestrian flows in the street space.

**TABLE 3:** Axis depth and connections of Huaihe Road Commercial Block.

Indicator name	Depth	Connection
Mean value	391.5	3.58
Minimum value	282	2
Maximum value	530	9
Standard deviation	53.9225	1.57

#### 3.2.2. Axis connections of Huaihe Road Commercial Block

As shown in Figure 3B and Table 3, the average connection of Huaihe Road Commercial Block is 3.58 and the axis within the block is reasonable in both cool color and warm color. For the main street of the block, the connection is 4–8 and the connection of the street space is 3–5, indicating that the main street has connected most of the street space in the block. The pedestrian street is the main street of the commercial block. As proven by the field investigations, the vitality of the main street is higher and the pedestrian flow of the main street is larger than that of the street space. As for the reason, pedestrians from different street space are gathered to the main street, increasing the vitality of the pedestrian street. In case of a peak pedestrian flow, pedestrians can be evacuated through the street space to alleviate congestion.

#### 3.2.3. Axis integration of Huaihe Road Commercial Block

Topological radius in space syntax is a key component of the space syntax model. Setting different topological radii in different spatial scenes facilitates a better understanding of urban spatial relationships. For the calculation of the axis integration of the Huaihe Road Commercial Block, the topological radius was set to be 3, 5, and n, and the axis integration R_3_, R_5_, and R_*n*_ under different topological radii was analyzed. As shown in Figure 3D and Table 4, the mean integration is 1.71 for R_3_, 1.39 for R_5_, and 1.26 for R_*n*_. Among the three topological radii, R_3_ has the highest mean integration, indicating that a smaller topological radius corresponds to higher spatial integration and better accessibility. The axis colors under the three topological radii are mostly warm colors, but some axis integrations tend to change and the integration of some roads in the block has improved. Rn is taken as an example in Figure 3D. According to this example, the axis of Huaihe Road Pedestrian Street is in warm color. The axis integration of the middle section of the main street is 1.92 while the integration of the east and the west entrance and exit is 1.19 and 1.47, demonstrating that the west section of the main street has stronger accessibility.

**TABLE 4:** Axis integration of Huaihe Road Commercial Block.

Indicator name	Value (*r* = 3)	Value (*r* = 5)	Value (*r* = n)
Mean value	1.71	1.39	1.26
Minimum value	0.95	0.89	0.83
Maximum value	2.65	1.93	1.92
Standard deviation	0.35	0.20	0.23

#### 3.2.4. Axis selection of Huaihe Road Commercial Block

For the calculation of the axis selection of Huaihe Road Commercial Block, the topological radius was set to be 3, 5, and n, and the axis integration R_3_, R_5_, and R_*n*_ under different topological radii was analyzed. As shown in Figure 3E and Table 5, the mean selection is 42 for R_3_, 180 for R_5_, and 300.5 for R_*n*_, proving that the mean selection of the block changes with the topological radius and the two factors are positively correlated. The range of variation is significant among the three selections. With the change in topological radius, the space is enlarged and the number of road axes in the space is increased, providing more optional routes and improving the mean selection. R_*n*_ is taken as an example in Figure 3D. The axis of the middle section in the main street is red with a selection of 2,171. However, the selection of other roads in the main street is not high. Some of the street spaces are connected with the entire block space in the form of road space, showing better selectivity.

**TABLE 5:** Axis selection of Huaihe Road Commercial Block.

Indicator name	Value (*r* = 3)	Value (*r* = 5)	Value (*r* = n)
Mean value	42	180.61	300.5
Minimum value	0	0	0
Maximum value	300	1,266	2,171
Standard deviation	54.42	235.34	395.43

#### 3.2.5. Axis intelligibility of Huaihe Road Commercial Block

This represents the relationship between the axis connection and global integration derived from the axis analysis. The axis intelligibility indicates the correlation of road space in the block. This indicator can reflect the importance of different roads in the block space. Higher axis intelligibility means that people can identify the urban road space more easily.

In respect of intelligibility, the connection is taken as the X axis and the global integration as the Y axis. If the value of intelligibility R^2^ is 0.5 to 1, it is considered that the connection is significantly correlated with global integration. According to Figure 3E, the axis intelligibility is 0.417, indicating that the correlation between the block connection and global integration is not high. Consumers can grasp the road space of the block through the local road space but will not get lost.

### 3.3. Spatial viewshed analysis of Huaihe Road Commercial Block

Viewshed analysis is a process to express the three-dimensional space analysis based on human perspective through different colors on a plane. The building profile map of the block is plotted using CAD software. Such analysis requires an overall enclosed space. Therefore, sealing lines are marked at the road exit and entrance of the block, and the viewshed analysis only focuses on the pedestrian flows in the block.

#### 3.3.1. Perspective connection of Huaihe Road Commercial Block

**FIGURE 4:**
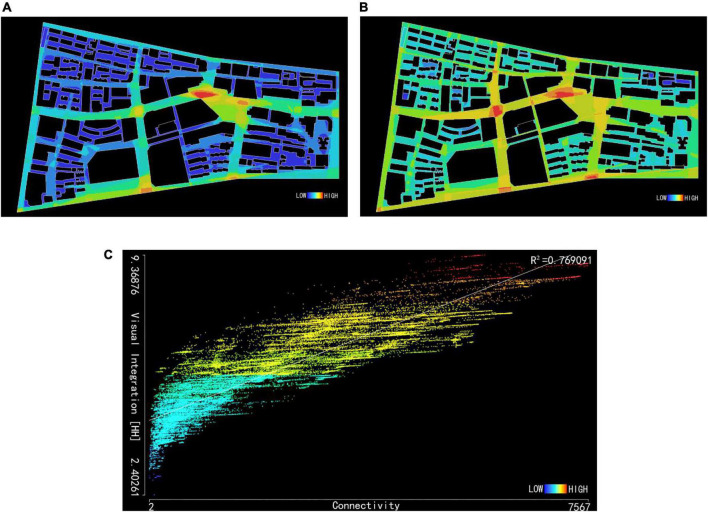
Perspective connection analysis of Huaihe Road Commercial Block **(A)**. Perspective integration analysis of Huaihe Road Commercial Block **(B)**. Perspective intelligibility analysis of Huaihe Road Commercial Block **(C)**.

Perspective connection analysis is a process to indicate the total number of elements seen in the perspective through colors. This indicator reflects the visibility of space around a specific location and also represents the changes in spatial scale and building density. As shown in Table 6 and Figure 4A, the mean perspective connection is 1,875.18, and most road intersections in the block are in cool color with a connection of 300–1,200, lower than the mean value because the building density is high at these locations and visions will be blocked by buildings so that people can only see fewer elements. However, the middle section of the pedestrian street is in warm color and the spatial connection is approximately 3,000–7,000, higher than the mean value because the pedestrian street is a belt-shaped street. Shops are distributed along the street without obvious obstructions. People can enjoy a better perspective at this location.

**TABLE 6:** Perspective connection values and perspective integration values of Huaihe Road Commercial Block.

Indicator name	Connection	Integration
Mean value	1,875.18	5.81
Minimum value	2	2.41
Maximum value	7,567	9.37
Standard deviation	1,514.49	1.14

#### 3.3.2. Perspective integration analysis of Huaihe Road Commercial Block

Block perspective integration analysis refers to the agglomeration or dispersion between a single element and other elements in the perspective to measure the ability of a space as a destination to attract traffic. It reflects the centrality of the space in the entire system. As shown in Table 6 and Figure 4B, the visual area of the block is in warm color and the mean perspective integration is 5.81. Its standard deviation is 1.14, tending to be balanced. The area with a high building density in the block is in a relatively cool color. The human perspective is blocked by dense buildings, resulting in poor integration in the area. The main street of the block is in warm color, with an appropriate spatial scale and better accessibility.

#### 3.3.3. Perspective intelligibility analysis of Huaihe Road Commercial Block

For the intelligibility of viewshed analysis, the connection is taken as the X axis and the global integration of perspective as the Y axis, and R^2^ is used to express the correlation between the two indicators, thus obtaining Figure 4C. Perspective intelligibility is an indicator to perceive the global spatial situation through local spatial information from human visions. The value of intelligibility R^2^ expressed by this area is greater than 0.5, indicating that the surrounding overall situation can be perceived through perspective. If the value is less than 0.5, the human ability to capture the overall spatial information through perspective is weakened accordingly. The intelligibility R^2^ of Huaihe Road Commercial Block is 0.769091, far greater than 0.5, indicating that local spatial information of the block can be recognized by the human perspective to perceive the block space. Dynamic pedestrian flows in different periods of the block can distinguish the block space through the line of sight. In particular, the overall space is grasped in the period with a peak pedestrian flow to alleviate the pedestrian flow.

### 3.4. Commercial forms of Huaihe Road Commercial Block

The distribution of commercial forms in the block was obtained by capturing the POI data of the block from relevant websites. According to the known distribution, the block has a complete range of commercial forms, involving ten forms, such as catering, shopping, life, accommodation, scenic spot, medical insurance, science, education and culture, transportation facility, finance and insurance, and sports and leisure. As shown in Table 7, the catering industry accounts for 47% of the total commercial forms. Consumer shopping ranks second, up to 22%, which differs greatly from the proportion of 4: 3: 3 (catering: shopping: entertainment) in commercial forms of traditional commercial streets. In this view, the block suffers unbalanced category and quantity ratios of commercial forms.

**TABLE 7:** Number of commercial form categories of Huaihe Road Commercial Block.

Category of functional commercial forms	Quantity
Catering service	1,355
Shopping service	638
Life service	513
Accommodation service	56
Scenic spot service	7
Medical insurance service	38
Science, education and culture service	96
Transportation facility service	54
Finance and insurance service	31
Sports and leisure service	85

### 3.5. Scenic spatial layout analysis of Huaihe Road Commercial Block

The deployment of scenic space can gather the popularity of pedestrian streets, increase the appreciation of landscape, and enhance the interactive experience of space. The renovation of the old pedestrian street calls for scenic space, which plays a decisive role in making the updated pedestrian street more competitive.

The scenic space of Huaihe Road Commercial Block mainly consists of four spatial forms, including leading space, evolutionary space, node space, and climactic space. Leading space refers to the exit and entrance of a commercial block. Evolutionary space refers to the linear space, such as main street and lane space, to guide consumers through the spatial path. Node space improves the single spatial form of linear space and brings changes in both spatial form and scale (Wang et al., 2015). Climactic space is the central part of a commercial block, which is formed by the square space of the block. Changes in its spatial scale can attract the attention of pedestrians. However, it should be combined with the corresponding scenic layout. Otherwise, it will be less attractive and pedestrians will not stop there (Sheng, 2012).

## 4. Research on the optimization strategy of Huaihe Road Commercial Block based on space syntax

### 4.1. Principles of optimizing the block space layout

#### 4.1.1. Principle of accessibility

The law of probability pioneered by Hough also proves the importance of accessibility for commercial blocks. According to the law, the probability for residents to go to a commercial block is determined by the commercial block scale and the distance to the commercial lock when the entire commercial block is centralized to a limited scope. Huaihe Road Commercial Block enjoys both good commercial scale and traffic accessibility within the old city of Hefei. Consumers prefer to select this block for consumption activities. Therefore, the optimization of the spatial layout for this block will not only the accessibility of block space but also improve the experience of consumers.

#### 4.1.2. Principle of identifiability

For a block, higher intelligibility corresponds to the people’s stronger perception toward space and also higher identifiability. Node space is an important space for a commercial block, which can optimize the human ability of spatial perception. The spatial form of commercial blocks is mostly belt-shaped pedestrian streets. Consumers will feel that the spatial form is too single. By changing the spatial scale, signs, and other ways, consumers will be guided to reach different positions, thus perceiving the overall spatial situation.

#### 4.1.3. Principle of selectivity

The more complex relationship of the block road network corresponds to the lower selectivity of each road for consumers. Consumers tend to choose the shortest path. The higher the path selection is, the better the road traffic attributes and potentials will be. Therefore, the roads with lower selection in the block should be repaired to improve the road quality.

### 4.2. Analysis of spatial layout optimization for Huaihe Road Commercial Block

#### 4.2.1. Traffic space optimization strategy

**FIGURE 5:**
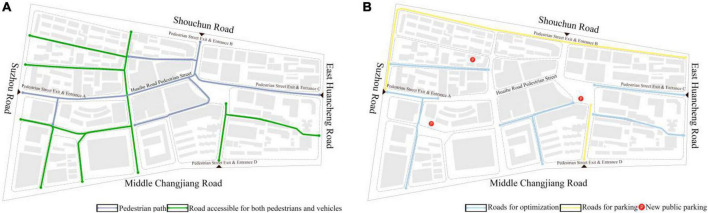
Current space of Huaihe Road Commercial Block **(A)**. Traffic space optimization of Huaihe Road Commercial Block **(B)**.

The traffic space of the block is divided into pedestrian space and vehicle space as shown in Figure 5A. Commercial pedestrian streets are special streets. Most of the roads in the block should be accessible for pedestrians only or both pedestrians and vehicles. The traffic space optimization of the block is analyzed according to Figures 3C, D. The block roads with lower integration and selection in the figures are optimized as shown in Figure 5B. These roads have a small spatial scale but many intersections. Consumers are prone to lose their sense of direction and have a poor spatial environment experience.

##### 4.2.1.1. Strategy of pedestrian street space optimization

Huaihe Road Commercial Block is a commercial block. The walk is the prevailing method of transportation in the block. Efforts should be made to improve the experience of consumers while walking on the block roads. Therefore, it needs to set road signs at road intersections, optimize road paving and street facade paving, and retreat some parts of some buildings in the street, thereby leading to changes in the spatial scale of streets and attracting consumers.

##### 4.2.1.2. Strategy of optimizing the space for carrying vehicle flow in the block

The problem of vehicle mobility and vehicle parking in the block is an urgent problem for the commercial block. Huaihe Road Commercial Block is located in the prosperous area of the old town. The overall accessibility of the block is strong and there will be a large vehicle flow. For this reason, it is recommended to widen the roads with poor accessibility and add landmark designs of trunk roads for traffic diversion, thus improving vehicle mobility in the block. It is also recommended to set public parking for the block around the space with no high integration and selection for traffic diversion or arrange temporary parking for the roads with lower selection to effectively carry more vehicle flows. In addition, this public parking will help to gather pedestrian flow. The tie between public parking and main street space will be strengthened, enabling the consumers to easily reach the pedestrian street. When doing so, the block will not only relieve the parking pressure but also realize the separation of pedestrians and vehicles, creating an excellent spatial environment for the commercial block.

#### 4.2.2. Strategy for optimizing scenic node space

**FIGURE 6:**
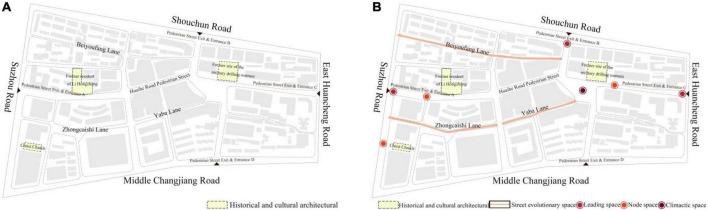
Scenic node space optimization of Huaihe Road Commercial Block **(A)**. Scenic node space optimization of Huaihe Road Commercial Block **(B)**.

Commercial blocks are mainly developed with pedestrian streets as the main axis and street space as the secondary axis. Refer to Figure 6A for details. A scenic node space is divided into leading space, evolutionary space, node space, and climactic space. By combining with the analysis in Figures 4A, B, the optimization of scenic node space for the block was analyzed. The optimization of the block space based on the different degrees of integration and connection in the viewshed diagram is shown in Figure 6B. The spatial environment experience of consumers in the block can be improved by optimizing and setting scenic node space.

##### 4.2.2.1. Leading space

The leading space is located at the exit and entrance of the pedestrian street in the block. The exit and entrances A, B, and C of the block shown in Figure 6A can be used as the leading space. The spatial perspective integration of these locations is not high. It is hard to attract consumers. These locations can hardly lead the consumers to the main street. It is accordingly proposed to enhance the diversity of the leading space, improve the leading space in terms of form, scale, and environment, strengthen the identification of the leading space, build up the cultural characteristics of the urban facade for Huaihe Road Commercial Block, and better impress consumers with the public space of the pedestrian street.

##### 4.2.2.2. Evolutionary space

The commercial space of Huaihe Road Commercial Block consists of the main street and street space. The associated streets and lanes of the block have a small spatial scale with lower perspective connection and integration. They can hardly attract consumers. Therefore, it is necessary to build up a characteristic cultural street space by combining with historical and cultural buildings of the block or with purposeful commercial forms to increase the commercial space of the block. In addition, the road quality of the street space should be optimized and sign boards should be set at the locations with higher perspective integration to guide the consumers and promote the vitality of the street space.

##### 4.2.2.3. Node space

The pedestrian street of Huaihe Road Commercial Block is a linear space, which will cause consumers to feel bored because the main street is too long. Moreover, the integration of such space is not balanced. Therefore, it is necessary to deploy some characteristic node spaces at the locations of the main street with lower integration and provide the space with green plants to constitute a scenic rest space, thereby creating a good external space environment for consumers.

##### 4.2.2.4. Climactic space

Climactic space is the central part of a commercial block. According to Figure 4B, the location with the highest perspective integration in Huaihe Road Commercial Block can gather a large pedestrian flow. The climactic space of Huaihe Road Commercial Block is an open square space, which has the highest spatial integration. Those pedestrian flows from the leading space and evolutionary space will gather in the climactic space. Therefore, the climactic space can be refined for diverse spatial functions, such as exhibition area, rest area, performance area, and temporary sales area, in an effort to retain consumers for the longest time and create an excellent commercial atmosphere for the pedestrian street.

#### 4.2.3. Strategy for optimizing the commercial space

**FIGURE 7:**
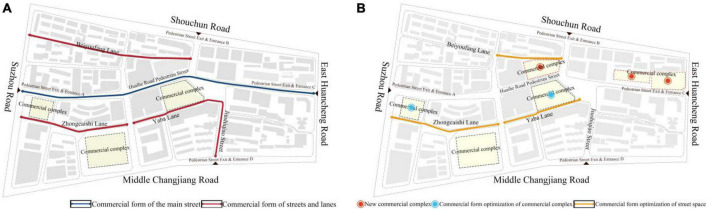
Current distribution of commercial space of Huaihe Road Commercial Block **(A)**. Commercial space optimization of Huaihe Road Commercial Block **(B)**.

Shops along streets and commercial complexes are the main commercial layouts of the Huaihe Road Commercial Block. Shops along streets were the prevailing commercial mode in the early stage of commercial blocks. With the development of commercial complexes, they are playing an indispensable role in commercial blocks. The quality of commercial forms will determine the commercial vitality of pedestrian streets. Major commercial forms for a pedestrian street are catering, shopping, and entertainment to improve the commercial quality and attract consumers (Sheng et al., 2014). According to Figure 4B and the analysis of commercial space optimization of the block based on the field investigations, the optimization of commercial space with higher integration in the block is recommended as shown in Figure 7B.

##### 4.2.3.1. Shops along streets

Shops along the streets of Huaihe Road Commercial Road are shown in Figure 7A. The main street has good perspective integration. As for the route, consumers prefer the exit and entrances A and C to enter the main street and complete consumption behaviors. They seldom focus on the commercial forms of street space. In this view, it is recommended to appropriately adjust commercial forms of the main street and associated streets and lanes and set more random consumption forms in the space of the main street such as clothing and footwear shopping services because the commodities in these commercial forms have a high price and a strong brand influence. They can help to improve the commercial quality of the pedestrian street. However, it is proposed to set purposeful consumption forms in the street space, such as bars, electronic game centers, and KTV. These consumption forms can easily attract specific consumers, boost the diversity of commercial forms in the block, and promote the vitality of street space.

##### 4.2.3.2. Commercial complex

The commercial complex has become the main commercial mode of commercial blocks in recent years. It is recommended to reasonably plan commercial complexes for locations with higher block integration (Lee, 2012). Commercial complexes are mostly located in the leading space and evolutionary space of the block as shown in Figure 7B. These locations have better perspective integration and connection and most consumers will pass through these locations. Therefore, a new commercial complex can be built in the leading space and climactic space to make full use of the high integration and set with such high-end commercial forms as catering, shopping, and entertainment to enhance the commercial value of the block. Furthermore, attention should be paid to shopping services while adjusting the commercial forms of a commercial complex in the leading space. Different commercial forms in the pedestrian street of Huaihe Road Commercial Block can also complement each other to promote consumption behaviors so that consumers will complete the activities in the pedestrian street to form a closed consumption streamline at the main exits and entrances of the block.

## 5. Conclusion

The spatial layout of Huaihe Road Commercial Block was studied in this article. The commercial block is an important public space in the daily life of residents. The optimization of the block’s spatial layout is a significant step forward for urban renovation. The following optimization strategies are obtained using the space syntax theory:

(1) The accessibility of roads within Huaihe Road Commercial Block to the old town of Hefei was obtained through space syntax analysis, thus explaining the location of commercial blocks at the city center.
(2) A clear understanding was obtained of the spatial layout of Huaihe Road Commercial Block through space syntax analysis, which is consistent with the facts discovered during field investigations.
(3) The corresponding strategies for optimizing the traffic space, scenic space, and commercial space of Huaihe Road Commercial Block were recommended by combining with the above space syntax analysis and the results of field investigations. For example, optimizations are recommended in the following aspects. In detail, the traffic space was optimized by changing the street space scale and setting sign boards to guide vehicle flows and reasonably arranging public parking; scenic space was optimized by improving the form, scale, and environment of the block space based on the spatial integration and visibility; the commercial space was optimized by setting appropriate commercial forms for the commercial complexes of the block based on the locations of these commercial complexes according to the block integration, commercial form, and pedestrian flow.

The above conclusions also make us aware that the optimization of the spatial layout of old blocks is an indispensable part of the current urban renovation. The combination of space syntax theory and block space design will contribute to the improvement of consumers’ consumption experience in commercial blocks.

## Data availability statement

The original contributions presented in this study are included in the article/supplementary material, further inquiries can be directed to the corresponding author.

## Author contributions

Both authors listed have made a substantial, direct, and intellectual contribution to the work, and approved it for publication.
